# Patient and surgical factors affecting procedure duration and revision risk due to deep infection in primary total knee arthroplasty

**DOI:** 10.1186/s12891-017-1915-4

**Published:** 2017-12-21

**Authors:** Mona Badawy, Birgitte Espehaug, Anne Marie Fenstad, Kari Indrekvam, Håvard Dale, Leif I. Havelin, Ove Furnes

**Affiliations:** 1 Coastal Hospital in Hagavik, 5217 Hagavik, Norway; 2 Center for Evidence-based Practice, Bergen University College, 5021 Bergen, Norway; 3 The Norwegian Arthroplasty Register, Department of Orthopaedic Surgery, Haukeland University Hospital, 5021 Bergen, Norway; 4 Department of Clinical Medicine, Institute of Medicine and Dentistry, University of Bergen, 5021 Bergen, Norway

**Keywords:** Knee, Osteoarthritis, Arthroplasty, Procedure duration, Infection, Risk factors, Revision

## Abstract

**Background:**

The aim of this study was to assess which patient and procedure factors affected both the risk of infection as well as procedure duration. Additionally, to assess if procedure duration affected the revision risk due to deep infection in total knee arthroplasty (TKA) patients and in a subgroup of low-risk patients.

**Methods:**

28,262 primary TKA with 311 revisions due to deep infection were included from the Norwegian Arthroplasty Register (NAR) and analysed from primary surgery from 2005 until 31st December 2015 with a 1 and 4 year follow up. The risk of revision due to deep infection was calculated in a multivariable Cox regression model including patient and procedure related risk factors, assessing Hazard Ratio (HR) with 95% confidence interval (CI).

**Results:**

Multivariate analysis showed statistically significant associations with revision due to deep infection and increased procedure duration for male patients, ASA3+ (American Society of Anesthesiologists) and perioperative complications. Procedure duration ≥110 min (75 percentile) had a higher risk of deep infection compared to duration <75 min (25 percentile), in the unadjusted analysis (HR = 1.8, 95% CI 1.3-2.5, *p* = 0.001) and in the adjusted analysis (HR = 1.5, 95% CI 1.0-2.1, *p* = 0.03). For low-risk patients, procedure duration did not increase the risk of infection.

**Conclusion:**

Male patients, ASA 3+ patients and perioperative complications were risk factors both for longer procedure duration and for deep infection revisions. Patients with a high degree of comorbidity, defined as ASA3+, are at risk of infection with longer procedure durations. The occurrence of perioperative complications potentially leading to a more complex and lengthy procedure was associated with a higher risk of infection. Long procedure duration in itself seems to have minor impact on infection since we found no association in the low-risk patient.

## Background

Numerous risk factors predispose patients to deep infection after total knee arthroplasty. It is critical to identify the correlation of risk factors that predispose TKA patients to deep infection, to reduce or even avoid this complication. Prolonged procedure duration has been demonstrated to increase the infection risk [1–5]. This is probably due to a combination of factors involving both the patient and the surgical environment, leading to bleeding and cautery, increased tissue damage and increased wound contamination.

Both surgeon and patient related factors can contribute to long procedure duration. Complexity of the surgery due to previous surgery to the knee or diagnoses other than primary osteoarthritis (OA) can increase procedure duration in addition to occurrence of perioperative complications. Inexperienced surgical team, low volume hospitals/surgeons could also contribute to longer procedure duration [3, 6]. Patient related factors increasing procedure duration are male sex, comorbidities, obesity and previous fractures around the knee [7, 8]. These factors are also well known risk factors of infection [1, 9–15].

The ‘Proceedings of the International Consensus Meeting on Periprosthetic Joint Infections’ by Javad Parvizi and Thorsten Gehrke [16] agrees with 96% delegate votes that surgical site infection rates increase directly with the duration of surgery. Their justification is numerous studies linking increased operative time to the risk of infection after total joint arthroplasty with statistical significance [1–3, 14, 17]. A study from Naranje et al. [12] demonstrated that operative time is only one of many factors that may increase infection risk and may be influenced by numerous confounders.

There are few reports on the relationship between long procedure duration and deep infection with revision as endpoint [1, 3, 12], and few describe the factors leading to prolonged procedure duration [8, 18].

Large study populations are required to measure rare events like deep infection. We used registry data [19] to determine risk factors for both prolonged procedure duration and deep infection and if there was an association between longer procedure duration and revision risk resulting from deep infection after TKA.

## Methods

**Fig. 1:**
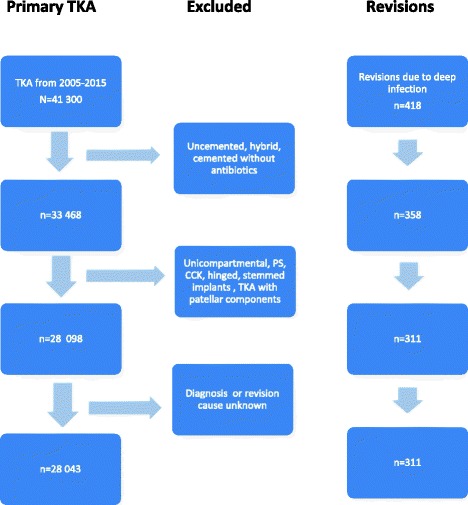
Flow chart with inclusion and exclusion criteria for total knee arthroplasties (TKA) reported to the Norwegian Arthroplasty Register from 2005 to 2015. Knees treated with uncemented, hybrid, cemented without antibiotics, unicompartmental, PS (posterior stabilized), CCK (constrained condylar knee), hinged, stemmed implants and TKA with patellar component were excluded for homogeneity reasons. Only TKAs with known operation time were included

TKA has been registered in the NAR since 1994. The completeness of reporting for primary procedures was 96% and 89% for revision surgery compared to data from the Norwegian Patient Registry [19]. In the present study, we included 28,262 primary TKA from 2005 to 2015. We selected the last 10 years of data to avoid outdated techniques and implants as well as less modern operating rooms. For homogeneity reasons, only cemented (with antibiotics) cruciate retaining (CR) implants (97% in the NAR) without patellar components (92% in the NAR) were included. Unicompartmental knee arthroplasty and more constrained implants were excluded (Fig. 1).

Revision was defined as complete or partial removal, exchange or addition of implant component(s). Patients with superficial wound infections treated with surgical site soft tissue debridement or with antibiotics only were not included in this study. A suggested follow-up time of 1 year would include all post-interventional infections thought to arise during implantation. Later there may be more haematogenous spread infections [20]. 1 and 4 years Kaplan Meier revision percentages are presented in Tables 3 and 4.

Procedure duration was recorded as the time from skin incision to complete wound closure in all cases. We used four different duration categories using quartiles (<75 min, 75-89 min, 90-109 min and ≥110). Data on patient related risk factors were collected; age, sex, comorbidity score (ASA (American Society of Anesthesiologists) classification), diagnosis and previous fractures or osteotomy to the knee. Hospital and surgery related risk factors were also collected; annual hospital volume, the occurrence of perioperative complications, the use of computer navigation (CAOS), implant brand and time period (Table 1).

**Table 1:** Patient and procedure characteristics at primary TKA relative to the four procedure duration groups

	Procedure Duration Groups				
	< 75 min	75 – 89	90 – 109	≥110	*p*-value
Number of procedures	5680	6238	8659	7685	
Year of operation 2010-2014 (*n* = 15,900) %	60	58	57	52	P < 0.001
Male sex (*n* = 10,186) %	28	31	37	44	P < 0.001
Age group %					P < 0.001
<60 (*n* = 4989)	15	17	17	21	
60-69 (*n* = 9717)	33	34	34	36	
70-79 (n = 10,009)	38	36	36	32	
≥80 (*n* = 3547)	14	13	13	11	
Median age (years) (range)	71 (31-96)	70 (25-94)	70 (23-101)	68 (22-93)	
Median annual hospital volume	118	113	95	86	P < 0.001
ASA %					P = 0.001
1 (*n* = 4167)	16	14	14	16	
2 (*n* = 17,918)	64	65	64	62	
3+ (*n* = 5621)	19	19	20	21	
Osteoarthritis (*n* = 25,152) %	92	90	88	87	P < 0.001
Perioperative complications (*n* = 640) %	0.7	1.1	1.8	4.9	P < 0.001
Previous surgery for intraarticular fracture or fracture near the joint (*n* = 551) %	0.8	1.3	1.7	3.6	P < 0.001
Previous high tibial osteotomy (*n* = 885) %	1.9	2.5	2.8	4.9	P < 0.001
Computer navigated TKA (*n* = 2462) %	2.5	5.6	9.0	18	P < 0.001
Systemic antibiotics (*n* = 28,108) %	100	100	100	100	*P* = 0.4
Prosthesis brand %					*P* < 0.01
LCS Complete (*n* = 8752)	26	31	31	34	
Profix (*n* = 6286)	23	24	23	20	
NexGen (*n* = 4717)	18	16	16	17	
AGC (*n* = 2233)	15	7.3	6.1	5.1	
Duracon (*n* = 2043)	5.7	6.8	6.8	9.2	
Triathlon (*n* = 1317)	5.2	4.0	5.2	4.1	
Vanguard (*n* = 741)	1.5	1.7	3.7	3.0	
PFC-Sigma (*n* = 697)	1.3	2.6	3.0	2.7	
LCS (*n* = 516)	1.3	1.2	2.0	2.5	
Other (*n* = 955)	3.3	5.0	2.9	2.7	

The majority of reported perioperative complications were different types of fractures, various tendon and ligament ruptures and technical issues regarding instruments or cementing, all increasing the probability of prolonged procedure duration.

Finally, a low-risk patient was defined based on the least probable risk of revision TKA from the analyses of all TKA presented in Tables 2 and 3; defined as a TKA patient with primary OA, classified as ASA 1 or 2, without any previous osteotomy or fracture to the knee and without any registered occurrence of perioperative complications.

**Table 2:** Patient and procedure related risk factors for revision due to infection after primary TKA

Variables	No	RR (95% CI) Unadjusted *p*-value		RR (95% CI) Adjusted	*p*-value
Age					
60-69	9717	1		1	
< 60	4989	0.9 (0.7-1.3)	0.7	0.9 (0.7-1.3)	0.6
70-79	10,009	0.8 (0.6-1.0)	0.1	0.8 (0.6-1.1)	0.1
> 80	3547	0.7 (0.5-1.1)	0.1	0.7 (0.5-1.1)	0.1
Sex					
men	10,186	1		1	
women	18,076	0.5 (0.4-0.6)	<0.001	0.5 (0.4-0.6)	<0.001
Diagnosis					
OA^a^	25,152	1		1	
Other^b^	3110	1.6 (1.2-2.1)	0.004	1.4 (1.0-2.0)	0.04
ASA					
1	4167	1		1	
2	17,918	1.1 (0.8-1.5)	0.7	1.2 (0.8-1.7)	0.4
3+	5621	1.7 (1.2-2.5)	0.005	1.8 (1.2-2.7)	0.003
Hospital volume					
1-49	3953	1		1	
50-99	10,615	1.1 (0.8-1.6)	0.5	1.1(0.8-1.6)	0.5
100-149	6379	1.2 (0.8-1.7)	0.4	1.1 (0.7-1.7)	0.6
≥ 150	7315	1.2 (0.8-1.7)	0.4	1.1 (0.7-1.7)	0.6
Perioperative complications					
no	27,068	1		1	
yes	640	2.3 (1.4-3.9)	0.002	2.1 (1.3-3.6)	0.004
Computer navigation					
no	23,626	1		1	
yes	2462	1.0 (0.7-1.5)	1.0	1.0 (0.7-1.5)	1.0
Prior fracture^c^					
no	27,711	1		1	
yes	551	2.20(1.1-3.6)	0.02	1.5 (0.8-2.7)	0.2
Prior osteotomy^d^					
no	27,377	1		1	
yes	885	0.9 (0.5-1.8)	0.8	0.8 (0.4-1.5)	0.5
TKA implant brands					
LCS Complete	8752	1		1	
AGC	2233	0.8 (0.5-1.3)	0.3	0.8 (0.5-1.3)	0.3
LCS	516	1.1 (0.5-2.3)	0.9	1.3 (0.6-2.9)	0.5
Duracon	2043	1.6 (1.1-2.3)	0.02	1.5 (0.8-2.7)	0.2
NexGen	4717	1.2 (0.8-1.6)	0.4	1.0 (0.7-1.4)	1.0
Profix	6286	0.9 (0.6-1.2)	0.4	0.9 (0.6-1.2)	0.4
PFC Sigma	697	1.0 (0.4-2.3)	1.0	0.9 (0.4-2.0)	0.7
Triathlon	1317	1.0 (0.6-1.8)	1.0	0.9 (0.5-1.5)	0.6
Vanguard TM	741	0.3 (0.1-1.0)	0.06	0.2 (0.1-0.8)	0.03
Others^e^	955	0.4 (0.1-1.0)	0.05	0.4 (0.1-1.0)	0.06
Time Period					
2005-2009	12,362	1		1	
2010-2014	15,900	1.3 (1.0-1.6)	0.03	1.3 (1.0-1.6)	0.08

^a^OA = Osteoarthritis

^b^Other = other diagnosis than osteoarthritis, e.g. inflammatory diseases

^c^Intraarticular fracture or fracture in proximity to the joint with previous osteosynthesis

^d^Previous knee osteotomy for knee malalignment

^e^Implant brands used in smaller numbers than 500 during the time period from 2005 to 2014

**Table 3:** Cox regression analysis. Risk of revision due to deep infection for all TKA patients in four different procedure duration groups

					Cox regression^c^			
					Unadjusted		Adjusted	
Procedure duration	No of TKA	No of revisions^a^	K-M 1y %^b^	K-M 4y %^b^	HR (95% CI)	*p*-value	HR (95% CI)	*p*-value
						0.01^d^		0.03^d^
<75	5680	48	0.60	0.89	1		1	
75-89	6238	54	0.58	0.85	1.1 (0.7 - 1.5)	1.0	1.0 (0.7 - 1.4)	0.9
90-109	8659	91	0.63	1.01	1.2 (0.9 – 1.7)	0.3	1.1 (0.8 – 1.6)	0.5
≥110	7685	118	0.91	1.38	1.8 (1.3 – 2.5)	0.001	1.5 (1.0 – 2.1)	0.03

^a^Number of revisions due to deep infection (*n* = 311)

^b^Kaplan-Meier estimated proportion of revisions due to deep infection at 1 and 4 years follow-up

^c^Unadjusted and adjusted Hazard ratios (HR) estimated with the Cox proportional hazards model (adjusted for sex, age, diagnosis, ASA classification and perioperative complications

^d^Overall test for group differences

### Statistics

Survival analyses were performed with first revision due to deep infection as endpoint. All cases were censored at December 31st 2015 to achieve at least 1 year follow-up for all primary TKA. Information about deaths and emigrations were obtained from the National Population Register. 1- and 4-year revision probabilities (time to revision due to deep infection) for the four procedure duration categories were calculated using the Kaplan-Meier method.

A Cox regression model was used to calculate the possible association between procedure duration and implant survival. Hazard ratios (HR) were represented with 95% confidence intervals (CI) and *p*-values relative to the shortest procedure duration as reference. All p-values less than 0.05 were considered statistically significant.

Both unadjusted (crude) and adjusted multivariate Cox proportional hazard models were used. Adjustment for potential confounding was performed. The model included common patient-related variables such as age, sex, diagnosis and ASA classification. The occurrence of perioperative complications were strongly associated with prolonged procedure duration and were therefore added to the adjustment.

**Fig. 2:**
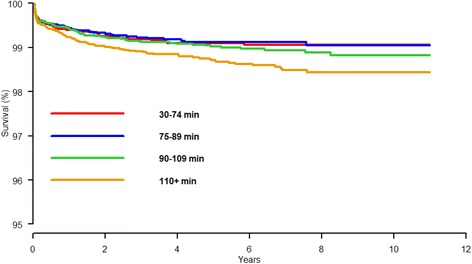
Cox regression survival curve for all included primary TKA with revision due to deep infection as endpoint for four different procedure duration groups. Adjusted for sex, age, diagnosis, ASA classification and perioperative complications

Similarly, unadjusted and adjusted Cox regression models were created for the low-risk patient previously described. Adjusted Cox regression curves were constructed for both models (Figs. 1 and 2).

The relative hazard assumption was tested by Schoenfeld residuals for chosen covariates and found to be valid. We found 13.3% bilateral procedures in our material and they were equally distributed in the infected and non-infected group. Death or emigration (lost to follow up) as a possible competing risk was investigated and there were no statistical significant differences in proportion of deaths within the groups, *p*-value equal to 0.15.

SPSS version 22 and R version 3.3.0 were used for the statistical analyses.

## Results

28,262 primary TKA were included for analysis and 311 patients underwent revision surgery for deep infection after TKA (1.1%) during the 11 year study period. Revisions due to infections accounted for 46% of all revisions within 1 year, and 27% within 4 years of follow up. Patient and surgery characteristics are presented in Table 1.

The mean and median procedure duration for non-infected cases was 94 and 90 min respectively, and for infected cases 100 min in both measures. The mean difference was statistically significant (*p* < 0.001).

Risk factors for prolonged procedure duration (≥110 min) were male sex, young age, diagnosis other than OA (inflammatory arthritis, OA due to previous fracture, ligament injury or infection), ASA 3+ patients, previous surgery to the knee, low hospital volume, perioperative complications, the use of CAOS, time period from 2005 to 2009 and implant brand (Table 1).

Adjusting for the other variables, males had a two times increased risk of revision resulting from deep infection as compared to females (p < 0.001). ASA 3+ patients had a 1.8 times higher risk of revision due to deep infection compared to patients classified as ASA 1 and 2 (*p* = 0.003). The occurrence of perioperative complications resulted in a 2.1 times higher risk of revision due to deep infection (*p* = 0.004) (Table 2).

The unadjusted Cox regression analysis showed statistically significant increased risk of revision resulting from infection comparing the longest duration group ≥110 min to the shortest procedure duration of <75 min by HR = 1.8 (95% CI 1.3-2.5, *p* = 0.001). (Table 3). After adjusting the Cox model for age, sex, diagnosis, ASA classification and the occurrence of perioperative complications, the effect of procedure duration was still statistically significant showing higher risk of revision due to deep infection in the longest duration group as compared to the shortest duration group; HR = 1.5 (1.0-2.1, *p* = 0.03) (Table 3, Fig. 2).

**Fig. 3:**
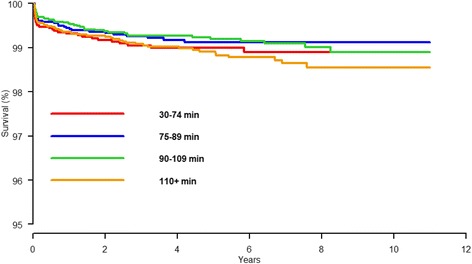
Cox regression survival curve for “low-risk” primary TKA patients with revision due to deep infection as endpoint for four different procedure duration groups with adjustment for age and sex. “Low-risk” TKA patient: patient with primary osteoarthritis, with ASA 1 or 2, without any previous surgery to the knee and no registered perioperative complications

Procedure duration did not influence the risk of revision due to infection in the low-risk patient (described in the methods section) neither in the crude (HR = 1.2, 95% CI 0.8-1.9, *p* = 0.3) or in the adjusted Cox regression analysis HR = 1.1, 95% CI 0.7-1.7, *p* = 0.6) (Table 4, Fig. 3).

**Table 4:** Cox regression analysis. Risk of revision due to deep infection for the low-risk patient^a^ in four different procedure duration groups

					Cox regression^d^			
					Unadjusted		Adjusted	
Procedure duration	No of TKA	No of revisions^b^	K-M 1y %^c^	K-M 4y %^c^	HR (95% CI)	*p*-value	HR (95% CI)	*p*-value
<75	3232	31	0.68	1.00	1		1	
75-89	3718	30	0.57	0.84	0.8 (0.5 - 1.4)	0.5	0.8 (0.5 - 1.3)	0.4
90-109	5130	44	0.49	0.78	0.9 (0.6 – 1.4)	0.6	0.8 (0.5 – 1.3)	0.4
≥110	4177	52	0.72	1.10	1.2 (0.8 – 1.9)	0.3	1.1 (0.7 – 1.7)	0.6

^a^The low-risk TKA patient: TKA patient with primary osteoarthritis, ASA 1 or 2, without any previous surgery to the knee and no registered perioperative complications (*n* = 16,257)

^b^Number of revisions due to deep infection (*n* = 157)

^c^Kaplan-Meier estimated proportion of revisions due to deep infection at 1 and 4 years follow-up

^d^Unadjusted and adjusted Hazard ratios (HR) estimated with the Cox proportional hazards model, adjusted for sex and age

## Discussion

Males, ASA 3+ patients, diagnosis other than OA and the occurrence of perioperative complications were factors associated with long procedure duration and increased risk of deep infection in this study (Table 2). In the low-risk patient we did not find evidence that increased procedure duration increased the risk of revision due to deep infection (Table 4). It could therefore be hypothesized that healthy patients that avoid perioperative complications tolerate longer procedure durations without getting infected.

Prolonged procedure duration may be caused by the complexity of the surgery and is thought to cause prolonged exposure time to microorganisms in the operating room and from the patient, possibly contaminating the wound. We found that risk factors for prolonged procedure duration was male gender, probably due to more difficult exposure related to extensor muscle mass and more dense bone cuts [12]. Similarly, young age, ASA 3+, previous surgery to the knee, low hospital volume, diagnosis other than OA and the use of computer navigation increased the procedure duration (Table 1).

There are several other publications on the effect of duration of surgery on deep infection; Namba et al. [1] conducted a subanalysis regarding duration of surgery and found a 9% increased risk per 15 min increment. Additionally, they found an increased risk of infection for male sex, ASA 3+ and other diagnoses than OA comparable to our results. However, perioperative complications as a confounding factor were not included in that study. Willis-Owen et al. found that the mean duration of surgery in non-infected patients was 102 (60-315) minutes versus 125 (80-201) minutes in the infected group. They did not, however, include confounding factors of comorbidities in their analysis [21]. Perioperative complications were not included as a variable in that study. They found an increased risk of infection in the >120 min group [22]. Naranje et al. [12] concluded that there was an effect of duration of surgery, but as one of many factors. Their conclusion was that after controlling for confounding variables, the effect of duration of surgery on risk of revision for infection was weak as an independent factor.

The strength of our study is the high number of primary TKA and the high completeness of registration in the NAR. Validation has found that 89% of all revisions after TKA were reported to the register from 2008 to 2012 [19]. However, there are some limitations to our study. The present study focuses solely on deep infection leading to revision of the knee arthroplasty either as debridement with exchange of the polyethylene bearing or as a complete 1- or 2-staged procedure. Some registry studies have shown underestimation of the incidence of reoperations due to infection [23]. A previous study on total hip arthroplasty from the Danish Hip Arthroplasty register, using multiple data sources, found nearly 40% underreporting of prosthetic joint infections [24]. The total number of deep infections in the present study is therefore probably underestimated. However, it is unlikely that the underreporting of infection cases is unevenly distributed among the duration groups.

Why males are more prone to revision for infection is probably multifactorial, but the sex difference has been studied. Male’s and female’s skin differ in hormone metabolism, hair growth and sebum production [25]. There have been demonstrated sex differences in skin pH and skin thickness that are possible factors for the differences in skin colonisation [26, 27] and thereby the increased risk of infection discussed in several studies [1, 12]. Our study found evidence to support that males are at higher risk of revision due to infection after TKA.

Infection rates in orthopaedic surgery are low and therefore causal factors are difficult to determine. Endogenous transmission of for instance Staphylococci carriers has also been shown to be an important cause of surgical site infection [28, 29]. Males have a higher carrier frequency of staphylococci which may partly explain their twofold risk of revision due to infection compared to women found in several studies [13, 14].

Perioperative complications resulted in prolonged duration of surgery and also risk of revision due to deep infection after TKA in our study. The majority of perioperative complications were different types of fractures, various tendon and ligament ruptures and technical issues regarding instruments and cementing. This highlights the importance of avoiding complications through education of surgeons and theatre staff, preoperative planning, good theatre routines and increasing volume of surgery. Perioperative complications might necessitate extended surgical approaches and added implants and devices could potentially harm the soft tissues, increasing the risk of hematomas, potentially increasing the risk of infection.

BMI (Body mass index) and other risk factors such as smoking or diabetes are not registered individually in the NAR, and is a limitation to this study. However, it is captured in the ASA classification. ASA classification has been shown to be a strong predictor of wound infection [30]. Increasing BMI is also a contributing factor to increasing duration of surgery [7] and some studies has found a correlation between increased BMI and postoperative infection after TKA [8, 9]. Others did not find similar relationship between obesity and infection [12]. Diabetes, irradiated skin, lymphedema, history of bleeding disorder could all lead to postoperative hematomas and wound-related problems and be associated with persistent wound drainage and deep infection [31, 32]. Implant brand affected procedure duration for two different implants (Table 1). The reason for this variety could be hospital and surgeon dependent, or that some implants require more steps in the procedure itself. However, implant brand did not affect the risk of deep infection.

## Conclusion

Male patients classified as ASA 3+, previous surgery to the knee and the occurrence of perioperative complications were factors requiring longer procedure duration and had a higher risk for infection after TKA in this study. Low-risk patients without perioperative complications did not have an increased risk of deep infection due to longer procedure durations. Long procedure duration in itself seems to have minor impact on infection since we found no association in the low-risk patient.

## Abbreviations

ASA: American Society of Anesthesiologists
BMI: Body mass index
CAOS: Computer assisted orthopaedic surgery
CI: Confidence interval
CR: Cruciate retaining
HR: Hazard ratio
K-M: Kaplan Meier
NAR: Norwegian Arthroplasty Røegister
OA: Osteoarthritis
TKA: Total knee arthroplasty
